# Balanced Identity in the Minimal Groups Paradigm

**DOI:** 10.1371/journal.pone.0084205

**Published:** 2013-12-31

**Authors:** Yarrow Dunham

**Affiliations:** Department of Psychology, Yale University, New Haven, Connecticut, United States of America; George Mason University/Krasnow Institute for Advanced Study, United States of America

## Abstract

Balanced Identity Theory [1] formalizes a set of relationships between group attitude, group identification, and self-esteem. While these relationships have been demonstrated for familiar and highly salient social categories, questions remain regarding the generality of the balance phenomenon and its causal versus descriptive status. Supporting the generality and rapidity of cognitive balance, four studies demonstrate that the central predictions of balance are supported even for previously unfamiliar “minimal” social groups to which participants have just been randomly assigned. Further, supporting a causal as opposed to merely descriptive interpretation, manipulating any one component of the balance model (group attitude, group identification, or self-esteem) affects at least one of the related components. Interestingly, the broader pattern of cognitive balance was preserved across such manipulations only when the manipulation strengthens as opposes to weakens the manipulated construct. Taken together, these findings indicate that Balanced Identity Theory has promise as a general theory of intergroup attitudes, and that it may be able to shed light on prior inconsistencies concerning the relationship between self-esteem and intergroup bias.

## Introduction

Theoretical work in social psychology frequently seeks to integrate phenomena that are otherwise studied separately. One recent effort in this regard, the Balanced Identity framework proposed by Greenwald and colleagues [1], builds on classic theories of cognitive consistency [2], [3], [4] by arguing that constructs such as attitudes, identification, and self-esteem reflect a coordinated set of associative relationships in semantic space. The framework makes formal predictions regarding how such constructs should relate, making it straightforward to test and extend in new directions. In particular, it provides a means of testing the ideas that intergroup bias emerges as a natural outgrowth of self-related positivity interacting with group identification.

**Figure 1:**
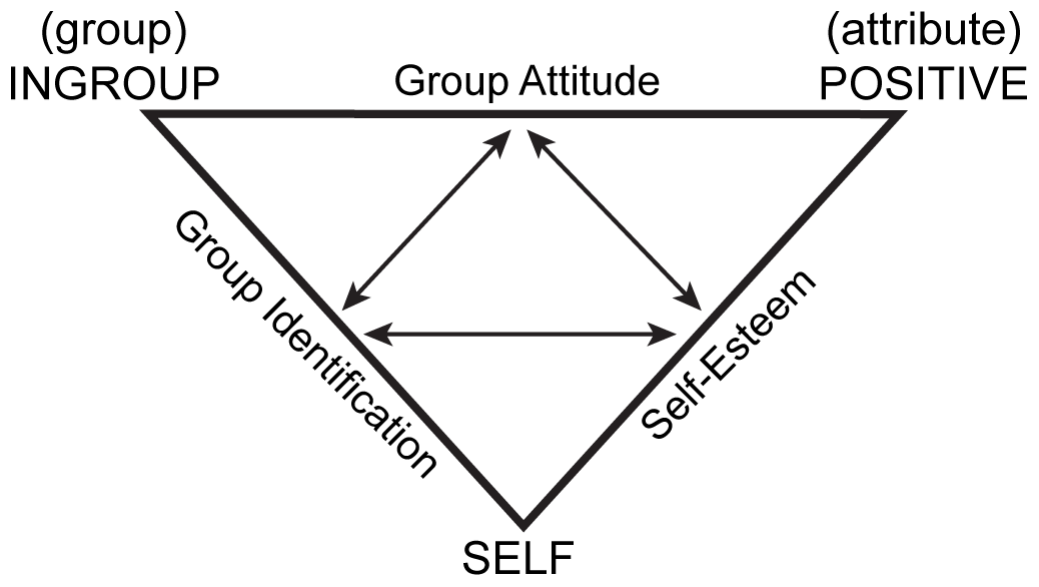
The Balanced Identity Framework. Vertices represent “nodes” in semantic space; outer edges represent the three principle constructs as associations between nodes. Internal arrows represent relationships between constructs, such that any one construct is (interactively) related to the other two. Figure adapted from [1].

More specifically, Balanced Identity entails the claim that ingroup preference must be understood in the context of several interrelated cognitive constructs, namely self-esteem and ingroup identification. These three constructs are conceptualized as occupying a triangular constellation of mutual associative influence (depicted in Figure 1). This cognitive structure allows a specific set of predictions to be made regarding the expected relationships among the constructs: any one construct can be predicted from the product of the other two. That is, one can predict the strength of a *group attitude* from the product of *self-esteem* and *identification* with that group, *self-esteem* from the interactive effect of *identification* and *attitude*, and so on. Because the model purports to describe patterns based on an associative memory system, it is suggested that these relationships will be most prevalent when constructs are themselves measured at the implicit or associative level, and will not necessarily apply to more explicitly held, propositional forms of information [1]. Recent meta-analytic evidence [5] provides strong support for the presence of these relationships across multiple attitude and stereotype domains.

So far, the Balance framework has been used to examine attitudes and stereotypes towards a range of groups [5], but primarily ones that are enduring and central to mature identity, such as gender and race/ethnicity. Capturing attitudes and identifications in these domains is a crucial test of model fitness, but because individuals have usually been members of these groups for a long time, we know very little about how the consistency processes postulated by Balanced Identity initially form. Is consistency the result of a protracted process of dissonance reduction in which inconsistencies are gradually reduced as related concepts are repeatedly co-activated? Alternatively, when a new attitude object (such as a social group) is encountered and evaluated, is the form of that evaluation immediately constrained by the strength and direction of existing cognitions (such as self-esteem), such that the new cognition is, from its genesis, in balance with its neighbors? Long-standing social affiliations cannot be used to address this question, because consistency observed long after their acquisition could be the result of either process. At present, the shortest time scale that has been investigated is about a week, in the specific case of attitudes towards residential colleges at a university [6].

The current research seeks to explore this problem space by asking whether attitudes and identifications learned just minutes before, within the context of the experimental setting, conform to the predictions of the Balanced Identity model. Participants are assigned to previously unfamiliar ‘minimal’ social groups [7], and self-esteem, (minimal) group attitude, and (minimal) group identification are assessed. If these cognitions conform to the predictions of Balanced Identity, it would favor the possibility that cognitive consistency emerges immediately, without requiring a gradual period of dissonance reduction. At a broader level, this inquiry can be considered a test of the generality of the Balance framework: Does it appear only with respect to highly familiar, personally important and culturally salient social groups, or can we observe it from the earliest moments of social affiliation, even with previously unfamiliar and minimally meaningful social groups?

The present work also addresses a second question regarding how the Balance Framework should be interpreted. All current investigations have been correlational designs, demonstrating that the posited pattern of relationships does exist with respect to real-world social groups. But the theory presumes to go beyond description; it posits that, for a given association, surrounding associations are, at least in part, *causally constitutive* of it. Testing this causal assumption requires moving beyond the correlational framework and directly manipulating constructs within the Balance framework to see if that manipulation affects neighboring constructs in the predicted directions. Thus, in addition to testing whether cognitive balance appears with newly encountered “minimal” social affiliations, in a follow-up series of experiments, each of the three “legs” of the Balance triangle (Figure 1) is manipulated independently in either the positive or negative direction, and the effect of that manipulation on related constructs is measured. This design allows us to test two corollary questions. First, does cognitive balance survive the direct manipulation of one of the cognitive constructs? An affirmative answer would again suggest that the balance relationships are rapidly emergent. Second and relatedly, several studies have suggested that balance does not emerge when the ingroup in question is less positively evaluated overall, for example when the ingroup is stigmatized, lower status, or more ambivalently viewed with respect to valence [6], [8], [9]. That raises the possibility that balance will be particularly disrupted when we manipulate constructs in the negative direction, e.g. by artificially reducing ingroup preference. Observing this in the present context would help to establish the generality of that previously observed phenomenon.

The Minimal Groups Paradigm (hereafter MGP) has established that participants consistently show preferences for previously unfamiliar, randomly assigned ingroups, and that these preferences occur across substantial methodological variation and on both self-report and implicit measures [10], [11], [12]. Because prior knowledge is controlled for through the novelty of the grouping dimension, the psychological consequences of “mere membership” can be directly assessed, providing a window into the generalized cognitive processes that underlie responses to group boundaries. This strategy has already proven valuable, showing, for example, that the tendency to show better recall for ingroup faces [13], to associate anger with outgroups [14], and to show signature neural responses to outgroup faces [15] are all general intergroup responses that emerge in similar fashion for highly familiar groups as well as minimal groups and that appear early in development [16], [17], suggesting that it is a reflection of core intergroup processes. In the current context, the question is whether the patterns of relationships described by Balanced Identity emerge outside the context of well-established and socially meaningful social collectives, i.e. in the minimal groups setting.

### Overview of the present research

In Experiment 1, participants are randomly assigned to a minimal social group, implicit and explicit group attitudes, group identification, and self-esteem are assessed, and the predictions of Balanced Identity regarding the relationships between the three constructs are tested. I predicted that cognitive balance would be present, supporting the possibility that Balanced Identity can serve as a general explanatory theory of intergroup bias, and that the relationships it describes emerge immediately and automatically in newly formed cognitions.

In Experiments 2–4, each of the three “legs” of the balance triad are manipulated prior to measurement. That is, each study experimentally manipulates one of group identification, group attitude, or self-esteem in either a positive (increasing strength) or negative (decreasing strength) direction. Manipulating all three constructs provides for an examination of the possibility that some of the links are more or less susceptible to manipulation than others. Most notably, at least within a minimal groups setting, self-esteem has a different status than identification with or attitudes towards a minimal group in that it is precedes the experimental setting. Indeed, positive implicit self-esteem is enduring, strong, and broadly present across persons and cultures [18]. By contrast, within the minimal groups setting, identification and attitude are emergent phenomena not supported by a long history of activation and/or reinforcement. This might make self-esteem more resistant to influence via manipulations of the others constructs, or it could make self-esteem more difficult to manipulate more generally and thus a less reliable inroad into affecting the other elements of the balance constellation. Thus, manipulating the identification with a social ingroup, for example, might be more likely to affect attitudes towards that group than self-esteem itself. Across these three experiments, I predicted that manipulations of one component of balance would affect the others, providing support for a causal reading of Balanced Identity.

## Experiment 1: Balanced Identity in the Minimal Group Paradigm

### Materials and Methods

#### Participants

All research reported in this paper was approved by the University of California, Merced Institutional Review Board. Ninety-seven participants, recruited from a university research study pool composed of undergraduate students participating for credit towards a course requirement in one of several psychology courses, participated in Experiment 1. Participants were highly diverse in terms of self-reported race/ethnicity (Asian = 40%, Hispanic = 33%, White = 19%, Black = 7%). Computer failure led to the elimination of data from seven participants prior to any analyses.

#### Procedure

Participants were greeted by a research assistant who secured written, informed consent and then escorted them to a private lab room. The participants were seated alone at a personal computer. The entire procedure took approximately 20 minutes, at which point they were debriefed and released.

#### Minimal group induction procedure

The experiment always began with the minimal group induction procedure, modeled after the recommendations provided by Pinter and Greenwald, who directly compared several minimal groups induction procedures [19]. Participants read a short paragraph indicating that the study involved two groups (the “Copley group” and the “Dawson group”), one of which they belonged to. They would next be introduced to the names of the members of their ingroup, which they had to learn in order to complete the later portions of the experiment. Participants then observed a static display listing the names of six members of their ingroup for 45 seconds. Following that, participants completed a name assignment task in which names appeared in the middle of the screen and had to be assigned to either the Copley or Dawson group by pressing one of two keys (the ‘e’ or the ‘i’ key), which were indicated on the screen next to the appropriate group label. For the initial 40 trials, participants were provided with a color cue indicating the group membership of each name, making categorization straightforward, but in a subsequent 30 trials, the color cue was removed so that participants would be encouraged to remember the names. Error feedback was presented when a name was miscategorized, and participants had to correctly categorize the name before continuing to the next trial. This procedure produced acceptable accuracy at group categorization (mean accuracy across the last 30 trials in all studies was greater than 93%). While participants always learned ingroup names first, the ingroup/outgroup name and the pairing of names to groups were counterbalanced across participants. This completed the minimal group induction procedure.

#### Measures

The primary dependent measures were three Implicit Association Tests [20]. The IAT is a dual categorization task in which, in the critical blocks, participants alternate between two categorization tasks involving four total target categories, but respond using only two response keys. The logic of the task is that if cognitively associated categories share a response key, responses will be facilitated, resulting in faster responding and less errors. For example, when categorizing positive words and words relating to the self with one key, and negative words and words relating to others with the other key, responding will generally be faster because of the cognitive congruence between positive words and a positively evaluated self. When the opposite pairings needs to be made (i.e., self paired with negative words, other paired with positive words), responding will generally slow down and the error rate will generally increase. The IAT is now the most well-validated measure of implicit attitude [21], [22].

The three IATs employed here were a group attitude IAT (contrasting Copley names and Dawson names as well as positive and negative adjectives), a group identification IAT (contrasting Copley and Dawson names as well as words relating to self and other, such as “me”, “my”, “I” versus “they”, “their”, and “them”), and a self-esteem IAT (contrasting self and other words as well as positive and negative adjectives). Because the strength of observed associations might depend on the temporal relationship between the IATs and the induction procedure, the two group-relevant attributes (attitude and identification) were measured prior to self-esteem. Thus, participants completed the attitude and identification IATs first (with task order counter-balanced across participants) followed by the self-esteem IAT. After the implicit measures, participants completed a short battery of self-report measures corresponding to the same three constructs of group attitude, group identification, and self-esteem. Following Pinter & Greenwald [19], these questions involved reporting on their liking for each of the two groups (e.g., “I like the Copley group”), their identification with each group (e.g. “I identify with the Dawson group”), and their liking for the self versus others (e.g. “I like myself”). To facilitate comparison with the IAT, difference scores were produced such that positive numbers indicate greater liking for and identification with the ingroup and greater liking for the self. The order of these measures was matched to the order of the IATs described above.

### Results

All data reported in these experiments are available online via the Open Science Framework, hosted at https://openscienceframework.org/project/Vvo9A.

#### Descriptive statistics

Following prior work with the IAT [23], response latencies greater than 10,000 milliseconds (ms) or less than 400 ms were dropped, and participants with excessive extremely fast trials (>10% of trials <300 ms) were dropped entirely; such participants are generally rapidly pressing buttons without responding to task instructions. These criteria led to the exclusion of four participants from the attitude and identity IATs and eight participants from the self-esteem IAT. Response time data were then used to calculate an effect size, the IAT *D* [26], reflecting the relative speed advantage in one condition. *D* was used in all analyses reported below, but for ease of interpretation we also include Cohen's *d* of the simple effects when reporting descriptive statistics. There were no main effects of group (Copley or Dawson), name-group pairing, or explicit group assignment on any dependent measures, so these factors were dropped from preliminary analyses. Beginning with the implicit measures, preference for the ingroup was robust, *D* = .29 (.36), *t*(85)  = 7.37, *p*<.001, *d* = .83, as was identification with the ingroup, *D* = .47 (.41), *t*(85)  = 10.50, *p*<.001, *d* = .93. Implicit self-esteem was strong and positive, *D* = .53 (.28), *t*(81)  = 17.23, *p*<.001, *d* = 1.18. Consistent with the notion that the MGP is essentially a manipulation of ingroup identification, identification as measured by the IAT was stronger than attitude as measured by the IAT, paired *t*(81)  = 3.56, *p*<.001. Preference for and identification with the ingroup were modestly correlated, *r*(80)  = .35, *p* = .001, but self-esteem correlated with neither identification, *r*(78)  = −.15, *p* = .18, nor preference, *r*(79)  = .05, *p* = .65.

Evidence for an effect of group membership was also present at the self-report level. Participants expressed more liking for the ingroup, *M* = .88 (1.7), *t*(89)  = 4.92, *p*<.001, *d* = .52, as well as greater identification with the ingroup, *M*  = 1.56 (2.1), *t*(89)  = 6.95, *p*<.001, *d* = .74 Participants also reported somewhat greater liking for the self as compared with others, *M* = .28 (1.2), *t*(89)  = 2.17, *p* = .03, *d* = .23. Correlations among self-report measures were consistently present, all *r*(88)>.28, *p*<.01. Strongest was the correlation between attitude and identification, *r*(88)  = .66, *p*<.001. Implicit and explicit measures did not correlate, all |*r*|<.18, *p*>.12.

#### Balanced Identity Analyses

Following the original formulation of Balanced Identity [1], evidence of balance is assessed for each of the three possible regression models created by predicting each construct from the other two. This involves first modeling each criterion's association strength from the product of the other two, and then in a second step entering the two predictors as main effects. The prediction is that the addition of these two main effect terms will not increase the predictive power of the model because the relationship is wholly accounted for by the product. This produces four tests that each model can be assessed against:

1. The regression coefficient associated with the interaction should be numerically positive and statistically significant at Step 1. A failure at this step indicates that the primary prediction of the theory has not been confirmed, and testing often ceases at this point. However, if this prediction is upheld then:
2. the coefficient associated with the interaction should remain numerically positive at Step 2 (after the main effect terms have been added);
3. neither regression coefficient associated with the main effect terms should statistically differ from zero at step 2; and
4. the increase in criterion variance (*R^2^*) at Step 2 should not be statistically significant.

Thus, these four tests can be used for each of the three regression models, providing 12 total tests that summarize the extent to which the predictions of cognitive balance are met in a given data set (independently for self-report and implicit data).

Results of these analyses are presented in Table 1 for both implicit and explicit data. For implicit data, evidence of balance was uniformly high, with all three models passing all four tests described above, except for one partial failure at Step 2 in which, for one model, one of the two main effect terms was statistically significant despite the presence (and continued statistical significance) of the interaction term. Nonetheless, 11 of 12 possible tests were passed, providing strong evidence of cognitive balance. For explicit results, evidence of balance was mixed, with all three models failing at least one test at Step 2 (in total, eight of 12 tests were passed). Thus, even though explicit measures were strongly correlated at the bivariate level (considerably more so than their implicit counterparts), they did not conform as well to the more complex pattern of relationships specified by the Balanced Identity model, replicating much prior work with this design [5].

**Table 1:** Summary of Balanced Identity Analyses for Experiments 1—4.

			Regression Step							Total tests passed
			Step1			Step 2				
		Criterion (*Y*)	Interaction^a^	*R^2^*	Interaction^b^		Main effect 1^c^	Main effect 2^c^	Δ *R^2^* d	
**Experiment 1**	**Implicit**	Attitude	.57***	.23	**.93**		**−.26**	**−.23**	**.03**	11/12
		Identification	.62***	.15	**1.00**		**−.27**	.34*	**.05**	
		Self-esteem	.33**	.08	**.44**		**−.14**	**−.05**	**.05**	
	**Explicit**	Attitude	.17***	.22	**.10**		.45***	**.05**	.29***	8/12
		Identification	.28***	.19	**.06**		.76***	**.00**	.25***	
		Self-esteem	.05***	.12	**.02**		**.20**	**.03**	**.02**	
**Experiment 2**	**Implicit**	Attitude	.46***	.11	**.37**		**.19**	**−.11**	**.03**	12/12
		Identification	.48***	.12	**.13**		**.26**	**.09**	**.03**	
		Self-esteem	.22*	.04	**.41**		**−.04**	**−.14**	**.01**	
	**Explicit**	Attitude	.20***	.27	**.06**		**.19**	.33***	.13***	7/12
		Identification	.36***	.24	**.11**		.61***	**.23**	.15***	
		Self-esteem	.07***	.18	**.04**		**.12**	**.09**	.06**	
**Experiment 3**	**Implicit**	Attitude	.70***	.16	**.50**		**.28**	**−.03**	**.02**	12/12
		Identification	.41***	.15	**.20**		**.13**	**.06**	**.01**	
		Self-esteem	.26**	.06	**.07**		**.12**	**.10**	**.01**	
	**Explicit**	Attitude	.11**	.07	**−**.05		**.07**	.54***	.31***	3/12
		Identification	.20***	.09	**−**.12		.81***	.56***	.34***	
		Self-esteem	.02*	.03	**−**.004		**−.003**	.16**	.06*	
**Experiment 4**	**Implicit**	Attitude	.31*	.06	**.35**		**−.10**	**.06**	**.01**	12/12
		Identification	.34*	.05	**.27**		**−.04**	**.13**	**.02**	
		Self-esteem	.41**	.08	**.11**		**.19**	**.14**	**.01**	
	**Explicit**	Attitude	.13***	.09	**.05**		**.05**	.37***	.28***	6/12
		Identification	.20*	.05	**.09**		.87***	**−.20**	.31***	
		Self-esteem	.01	.01	**−**.01		**.19**	**−.05**	**.01**	

Notes: Alpha levels are * *p*<.05, ** *p*<.01, *** *p*<.001.

^a^ Should be statistically significant and positive in order to past test.

^b^ Should remain numerically positive in order to pass test.

^c^ Should both not differ statistically from zero in order to pass test.

^d^ Should not be statistically significant in order to pass test. Cells in bold represents results consistent with predictions of the Balanced Identity model.

### Discussion

Upon inducing associations between participants and one of two novel groups, robust preference for and identification with the ingroup emerged, especially when measured at the implicit level. These findings add another replication to the large body of work employing the MGP. The novel contribution, however, is the demonstration that the predictions of Balanced Identity are satisfied by emergent cognitions in the MGP and therefore need not rely on an iterative or otherwise protracted period of enculturation or experience.

The fact that new group-related attitudes and identifications formed in such a way as to be in balance with pre-existing associations (i.e., self-esteem) suggests that the Balanced Identity relationships can thought of as causal determinants of the strength and direction of newly formed associations such as group attitudes. The next set of studies directly tests causality by manipulating each construct and measuring the effect of this manipulation on related constructs.

## Experiment 2: Manipulating Group Identification

This experiment follows a nearly identical procedure to that described in Experiment 1, except that, following group assignment, the strength of the associative self-group relationship is manipulated. Because Balanced Identity emerges so much more consistently at the implicit level, a manipulation of group identification that also targeted associations (as opposed to the explicit reporting of a self-group relationship) was employed. The central question, then, was whether manipulating identification will affect group attitude and/or self-esteem, and what effect, if any, this will have on the emergence of cognitive balance. This experiment also begins to explore a secondary question. Some prior work has suggested that balance does not emerge as reliably for members of lower status groups, such as members of less prestigious residential colleges [6] or ethnic minorities [8]. This pattern can be formalized as the prediction that balance will not be as robust for groups that are less positively evaluated. The current manipulation involved artificially increasing group identification in half the participants, and artificially decreasing it in the other half. If the phenomenon described above is general, we could see a disruption of balance specifically in individuals in the latter condition.

### Methods

#### Participants

One hundred twenty-two participants were recruited from the same population and following the same procedure described in Experiment 1.

#### Procedure

The procedure was closely modeled after that employed in Experiment 1; participants were assigned to groups in the same way as described there. They completed a manipulation of group identification followed by the same dependent measures as in Experiment 1.

#### Manipulation of group identification

The self-group association was manipulated using a partial-IAT procedure [10]. Participants were told that they were to imagine that they had the opportunity to spend a large amount of time with one of the two groups (the ingroup or the outgroup, as a between-participants factor). To simulate this experience, they would perform a categorization task in which they would categorize words related to the self with members of that group and words related to others with members of the other group. They completed two blocks of 60 trials in which they either repeatedly responded to self and ingroup names using one key and other and outgroup names using another key, or repeatedly responded to self and outgroup words using one key and other and ingroup words using another key. Thus, as a between-participants factor, the manipulation was designed to either bolster the self-ingroup relationship or weaken that relationship while bolstering the self-outgroup relationship. To avoid associating any response with a particular side of the screen, the pairing of group and self was counterbalanced across the two blocks of trials.

#### Measures

The same measures used in Experiment 1 were used here, namely implicit and explicit measures of group attitude, group identification, and self-esteem.

### Results

#### Descriptive statistics

Standard exclusion criteria for IAT results led to the elimination of data from 10 participants from the attitude and identity IATs and 16 participants from the self-esteem IAT. There was one significant effect of ingroup name (Copley or Dawson), with stronger ingroup preference for participants assigned to the Copley group, *t*(109)  = 2.84, *p* = .005. However, because this was the only effect of group name across the four experiments reported here and because it did not interact with other reported findings, it is not interpreted further.

Overall patterns of results were highly similar to Experiment 1, with participants exhibiting robust preference for the ingroup, *D* = .30 (.37), *t*(110)  = 8.34, *p*<.001, *d* = .78, robust identification with the ingroup, *D* = .49 (.36), *t*(111)  = 14.42, *p*<.001, *d* = 1.06, and strong implicit positive self-esteem, *D* = .47 (.30), *t*(103)  = 15.91, *p*<.001, *d* = 1.18. As in Experiment 1, identification was stronger than attitude, paired *t*(108)  = 4.65, *p*<.001. Preference for and identification with the ingroup were modestly correlated, *r*(107)  = .35, *p*<.001, but again self-esteem as not correlated with attitude, *r*(101)  = .13, *p* = .21, or identification, *r*(102)  = .13, *p* = .20. Evidence for an effect of group membership was also present at the self-report level. Participants expressed more liking for the ingroup, M = .70 (1.7), *t*(121)  = 4.50, *p*<.001, *d* = .41, as well as greater identification with the ingroup, M  = 1.3 (2.4), *t*(121)  = 5.95, *p*<.001, *d* = .54. Participants also reported somewhat greater liking for the self as compared with others, M = .47 (1.2), *t*(121)  = 4.39, *p*<.001, *d* = .39. Correlations among self-report measures were moderate, all *r*(120) >.39, *p*<.001. Strongest was the correlation between attitude and identification, *r*(120)  = .59, *p*<.001. Implicit and explicit measures did not correlate, all |*r*|<.11, *p*>.25.

#### Effect of identification manipulation

As noted above, half of participants completed a manipulation designed to strengthen the self-group relationship (the “match” condition), while the other half completed a manipulation designed to weaken it (the “mismatch” condition). The manipulation was effective; participants in the match condition exhibited markedly stronger ingroup identification as measured by the IAT, *M*_MATCH_ = .70 (.27), *M_MISMATCH_* = .29 (.32), *t*(110)  = 7.38, *p*<.001, *d* = 1.4, though identification remained positive and significant even in the mismatch condition, *t*(57)  = 6.98, *p*<.001. This manipulation also affected ingroup attitude, *M*_MATCH_ = .37 (.37), *M_MISMATCH_* = .22 (.39), *t*(109)  = 2.20, *p* = .03, *d* = .41, but did not affect self-esteem, *M*_MATCH_ = .49 (.27), *M_MISMATCH_* = .45 (.33), *t*(102)  = .61, *p* = .54, *d* = .13. The manipulation had no effect on any of the explicit measures, all *t*<.72, *p*>.47.

#### Balanced Identity Analyses

Table 1 provides a summary of results of the Balanced Identity analyses for implicit and explicit data, collapsing across condition. Overall, evidence for balance was strong at the implicit level but weak at the explicit level, with 12 and 7 tests passed, respectively. To examine whether the relative degree of balance differed depending on experimental condition, and in particular whether balance was less robust in the mismatch condition in which identification was reduced, these analyses were also conducted separately for each condition. However, one potential limitation of such an approach must be noted. By intervening on group identification in either the positive or negative direction, we may have artificially reduced the variability in that measure, which would work against finding evidence of balance on statistical rather than conceptual grounds. Thus, the following analyses should be interpreted with this in mind and in the context of supplementary analyses presented in the next section.

Table 2 provides these analyses sub-divided by condition (match or mismatch). However, when broken down by condition the results are more nuanced. Beginning with the implicit data, evidence for balance was once again strong in the match condition, with 12 out of 12 tests successfully passed. The results were quite different in the mismatch condition. All three models failed at Step 1, indicating that the interaction did not predict the criterion, nor did it do so at Step 2 when main effects were included. Thus, there was no evidence of balance when group identification was manipulated in the negative direction. The results from explicit data, while not providing strong evidence of balance, were relatively consistent across the two conditions, with 8 of 12 total tests passed for data from the match condition and 6 of 12 from the mismatch condition.

**Table 2:** Summary of Balanced Identity Analyses for Experiment 2, sub-divided by condition.

			Regression Step							Total tests passed
			Step1			Step 2				
		Criterion (*Y*)	Interaction^a^	*R^2^*	Interaction^b^		Main effect 1^c^	Main effect 2^c^	Δ *R^2^* d	
Experiment 2 (**Match Condition**)	**Implicit**	Attitude	.40**	.14	**.36**		**.22**	**−.08**	**.03**	12/12
		Identification	.38**	.14	**.30**		**.13**	**−.04**	**.02**	
		Self-esteem	.35*	.10	**.69**		**−.10**	**−.29**	**.01**	
	**Explicit**	Attitude	.10***	.25	**.05**		**.07**	.65***	.52***	8/12
		Identification	.07*	.10	**.15**		.75*	**−**.85*	.48***	
		Self-esteem	.04**	.14	**.04**		**.29**	**.31**	.06**	
Experiment 2 (**Mismatch Condition**)	**Implicit**	Attitude	.39	.03	**.17**		**.19**	**.08**	**.01**	7/12
		Identification	.28	.05	**−**.03		**.18**	**.26**	**.04**	
		Self-esteem	.13	.01	**−**.15		**.23**	**.03**	.**04**	
	**Explicit**	Attitude	.05*	.08	**−**.04		.46***	.**21**	.31***	6/12
		Identification	.10**	.16	**−**.06		.84***	**.56**	.34***	
		Self-esteem	.05**	.14	**.10**		**−.35**	**−.16**	**.03**	

Notes: Alpha levels are * *p*<.05, ** *p*<.01, *** *p*<.001.

^a^ Should be statistically significant and positive in order to past test.

^b^ Should remain numerically positive in order to pass test.

^c^ Should both not differ statistically from zero in order to pass test.

^d^ Should not be statistically significant in order to pass test. Cells in bold represents results consistent with predictions of the Balanced Identity model.

#### Supplementary analysis: Comparison with Experiment 1

**Figure 2:**
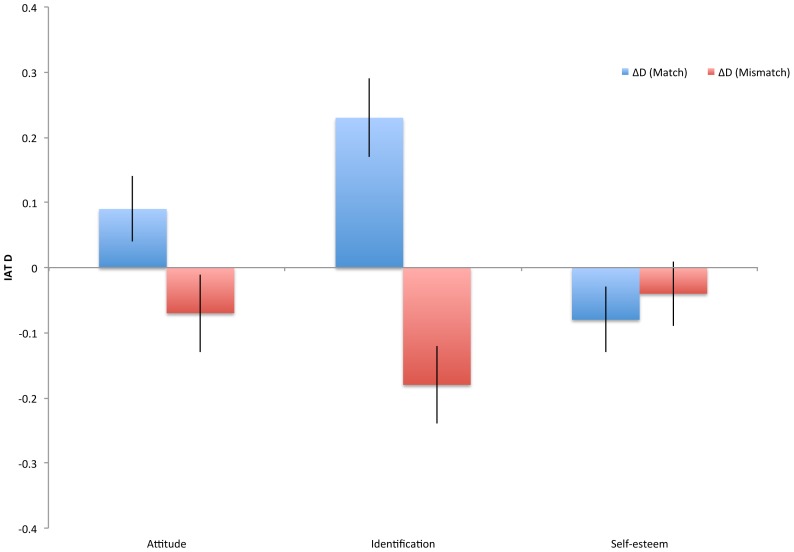
Difference between mean values of Experiment 1 and mean values of the match and mismatch conditions of Experiment 2. Error bars represent standard error of the mean difference; units are the IAT effect size *D*.

Because our design did not feature a control group for whom attitudes were not manipulated, the relative degree of change from baseline in the match versus mismatch condition is difficult to ascertain. However, Experiment 1 resembles such a control group, in that the design was identical to Experiment 2 except there was no manipulation of the identification construct. Indeed, the means across these two experiments (collapsing cross condition in Experiment 2) were highly similar, and did not differ significantly, all *t*<1.37, *p*>.17. Thus, under the assumption that the means in Experiment 1 are a reliable estimate of what we would have observed in Experiment 2 if there had been no manipulation, we can gain some insight into whether one condition was a more powerful elicitor of change by comparing Experiment 1 to the two conditions of Experiment 2. We can also use this analysis to explore the issue of reduced variability described above by comparing variances directly to see if variation was reduced with respect to any of the constructs. Results are presented in Figure 2, showing the average change and associated standard errors for the comparisons between Experiment 1 and the match and mismatch conditions of Experiment 2. The results suggest equivalence in the strength of the positive and negative manipulation; i.e., it does not appear to have been easier to increase or decrease identification and attitudes.

Supporting the worry that the manipulation might have reduced variance, the null hypothesis of equal variance was rejected in only the two cases in which ingroup identification in Experiment 1 was compared with ingroup identification in the match and mismatch conditions of Experiment 2. As expected, in both cases variability was lower in the Experiment 2 conditions. However, this finding does not appear able to explain the lack of balance found in the mismatch condition as compared to the match condition. This is because while variance was reduced relative to Experiment 1, it was reduced equivalently in the match and mismatch conditions, in which the null hypothesis of equal variance could not be rejected, *F*(57, 53)  = 1.39, *p* = .23. Thus, the differential degree of balance cannot be attributed to reduced variability in one of the criterion constructs.

### Discussion

In Experiment 2, identification with a minimal ingroup was manipulated by either weakening or strengthening the associative relationship between self and group in a partial-IAT procedure. This manipulation was successful and constituted a large effect; participants in the “mismatch” condition were much less implicitly identified with their ingroup. This manipulation did not, however, affect self-reported identification, and participants continued to explicitly identify with their ingroup. This demonstrates that the manipulation did not simply *confuse* participants about their membership, but rather affected the associative relationship between self and group. Importantly, the manipulation of identification also affected implicit attitude, with participants in the mismatch condition showing weaker implicit preference for their ingroup. Thus, manipulating one leg of the “Balance triangle” exerts causal influence on at least one of its neighbors, lending support to a causal rather than merely descriptive interpretation of these relationships. It is interesting that implicit self-esteem was not similarly affected. In general, a causal interpretation of Balanced Identity merely predicts that changing one construct will change related constructs so as to preserve balance; it is agnostic with respect to exactly *how* the equation is balanced. That is, when group identification changes, the cognitive system could respond by adjusting self-esteem, group attitude, or both. In this case, the predominant operation was to decrease the strength of group attitude. A plausible interpretation of this pattern of results is that because the self-positive association precedes the experimental setting and generalizes far beyond it, it is more resistant to minor perturbations via associated constructs of the sort created here. More leverage will be gained on this issue after manipulating the other constructs and describing their downstream effects.

Interestingly, the mismatch condition, which reduced group identification (and consequently group attitude), also disrupted balance; tests of balance in this condition all failed at the first step. By contrast, participants who had their group identification increased through the match manipulation continued to show robust balance, passing all 12 tests. This finding supports past work contending that belonging to a low status or otherwise non-liked group prevents cognitive balance, though the present findings allow us to generalize this considerably because “low status” in this case in no way reflects richer conceptions of enculturation but rather the results of a simple associative manipulation designed to reduce the self-group association. Supplementary analyses also suggested that the failure to find balance in the mismatch condition cannot simply be attributed to reduced variance in one of the constructs. The implications of this finding will be discussed further as data from the other experiments is presented. More broadly, however, we again saw clear evidence of balance on implicit but not on explicit measures.

## Experiment 3: Manipulating Group Attitude

This experiment followed a nearly identical procedure to that described in Experiment 2, except that group attitude was manipulated instead of group identification. In one direct sense, the MGP is in fact a manipulation of group identification; that is, it attaches participants to a group. By contrast, it does not provide evidence directly relevant to attitude or self-esteem. Thus, the manipulation in Experiment 2, above, could have been effective because it directly targeted the same factor implicated in the minimal group paradigm itself. If so, the causal effects found in that experiment could be thought of as simply strengthening or weakening the minimal group induction itself, rather than a consequence of causal relationships specified by the Balanced Identity model. However, if those effects were in fact the result of the consistency-generating processes described by Balanced Identity, results should be similar if another leg of the Balance triangle (in this case, group attitude) is manipulated. The central question, then, is whether manipulating attitude towards the newly assigned minimal group will affect the other two related constructs. A secondary question is whether artificially decreasing group attitude through our manipulation will disrupt the formation of cognitive balance.

### Methods

#### Participants

One hundred thirty-five participants were recruited from the same population and following the same procedure described in Experiments 1 and 2.

#### Procedure

The procedure was closely modeled after that employed in Experiment 2, except that the manipulation targeted attitude instead of identity.

#### Manipulation of group attitude

As in Experiment 2, a partial-IAT procedure was employed. Participants again completed two blocks of 60 trials in which, as a between-participants factor, they either repeatedly responded to ingroup and positive words using one key and outgroup and negative words using another key, or repeatedly responded to outgroup and positive words using one key and ingroup and negative words using another key. Thus, as a between-participants factor, the manipulation was designed to either increase or decrease the positive association with the ingroup (and affect the positive association with the outgroup in the opposite direction).

#### Measures

The same measures used in Experiment 1 were used here.

### Results and Discussion

#### Descriptive statistics

Standard exclusion criteria for IAT results led to the elimination of data from nine participants from the identification IAT and 11 participants from each of the other IATs. There were no effects of ingroup name or task order, and so these factors were dropped from subsequent analysis. Overall results again mirrored those described in the prior experiments, with participants exhibiting robust preference for the ingroup, *D* = .28 (.42), *t*(123)  = 7.60, *p*<.001, *d* = .68, robust identification with the ingroup, *D* = .48 (.29), *t*(125)  = 18.45, *p*<.001, *d* = 1.26, and strong implicit positive self-esteem, *D* = .51 (.31), *t*(123) = 18.33, *p*<.001, *d* = 1.37. Identification was again stronger than attitude, paired *t*(123)  = 18.33, *p*<.001. As in prior studies, implicit identification with the ingroup was stronger than implicit preference for the ingroup, paired *t*(122)  = 5.21, *p*<.001. Turning to correlations between constructs, preference for and identification with the ingroup were modestly correlated, *r*(121)  = .38, *p*<.001, and weaker correlations were observed between attitude and self-esteem, *r*(120)  = .23, *p* = .01, and identification and self-esteem, *r*(121)  = .19, *p* = .04.

Participants self-reports also revealed more liking for the ingroup, M = 1.0 (1.8), *t*(134)  = 6.43, *p*<.001, *d* = .56, as well as greater identification with the ingroup, M  = 1.8 (2.2), *t*(134) = 9.29, *p*<.001, *d* = .82. Participants also reported somewhat greater liking for the self as compared with others, M = .50 (1.1), *t*(134)  = 5.18, *p*<.001, *d* = .45. Correlations among self-report measures varied from weak in the case of self-esteem and attitude, *r*(133)  = .17, *p* = .051, moderate for self-esteem and identification, *r*(133)  = .30, *p*<.001, and strong in the case of attitude and identification, *r*(133)  = .61, *p*<.001. Implicit and explicit measures did not correlate, all |*r*|<.15, *p*>.10.

#### Effect of attitude manipulation

Half of the participants completed a manipulation designed to strengthen preference for the ingroup (“match” condition) while the other half completed a manipulation designed to weaken it (“mismatch” condition). This manipulation was effective; participants in the match condition exhibited markedly stronger ingroup attitude as measured by the IAT, *M*_MATCH_ = .56 (.31), *M_MISMATCH_* = .03 (.32), *t*(122)  = 9.29, *p*<.001, *d* = 1.66. Indeed, participants in the mismatch condition no longer preferred their ingroup following this manipulation, *t*(64)  = .74, *p* = .45. This manipulation also affected ingroup identification, *M*_MATCH_ = .58 (.29), *M_MISMATCH_* = .39 (.27), *t*(124)  = 3.93, *p*<.001, *d* = .68, though identification remained positive and significant in the mismatch condition, *t*(64)  = 11.61, *p*<.001. This time the manipulation also affected implicit self-esteem, *M*_MATCH_ = .59 (.30), *M_MISMATCH_* = .44 (.31), *t*(122)  = 2.91, *p* = .004, *d* = .49. The manipulation had no effect on explicit measures, all *t*<1.32, *p*>.19.

#### Balanced Identity Analyses

Table 1 provides a summary of results of the Balanced Identity analyses for implicit and explicit data, collapsing across condition; again, balance was clearly observed in the implicit but not explicit data. Table 3 provides these analyses sub-divided by condition (match or mismatch). Again results suggest that balance was disrupted in the mismatch condition. For implicit data in the match condition, evidence for balance was strong, with 10 out of 12 tests successfully passed, though it is important to note that one model (with self-esteem as the criterion) failed to show a significant interaction at Step 1, an important deviation from predictions. At the explicit level, evidence of balance was completely absent, with all models failing all tests. For participants in the mismatch condition all three models focusing on implicit data failed at Step 1, indicating little support for balance. For explicit data, there were some hints of balance, though not definitive, with 8 of 12 tests passed successfully.

**Table 3:** Summary of Balanced Identity Analyses for Experiment 3, sub-divided by condition.

			Regression Step							Total tests passed
			Step1			Step 2				
		Criterion (*Y*)	Interaction^a^	*R^2^*	Interaction^b^		Main effect 1^c^	Main effect 2^c^	Δ *R^2^* d	
Experiment 3 (**Match Condition**)	**Implicit**	Attitude	.39*	.10	**.63**		**.15**	**−.39**	.10	10/12
		Identification	.48***	.20	**.33**		**.15**	**−.01**	**.01**	
		Self-esteem	.14	.02	**.14**		**.12**	**−.10**	**.01**	
	**Explicit**	Attitude	**−**.03	.01	**−**.16		.57***	**.02**	.45***	0/12
		Identification	.02	.00	**−**.27		.84***	.93***	.53***	
		Self-esteem	**−**.02	.01	**−**.05		**−.00**	.25**	.18***	
Experiment 3 (**Mismatch Condition**)	**Implicit**	Attitude	.18	.01	**−**.29		**.22**	**.21**	**.01**	6/12
		Identification	.07	.00	**−**.17		**.16**	**.06**	**.02**	
		Self-esteem	.09	.00	**−**.10		**.06**	**.13**	**.01**	
	**Explicit**	Attitude	.24***	.32	**.06**		**.16**	.47***	.16***	8/12
		Identification	.34***	.32	**.15**		.60***	**−.07**	.16***	
		Self-esteem	.06***	.21	**.07**		**−.01**	**.03**	**.00**	

Notes: Alpha levels are * *p*<.05, ** *p*<.01, *** *p*<.001.

^a^ Should be statistically significant and positive in order to past test.

^b^ Should remain numerically positive in order to pass test.

^c^ Should both not differ statistically from zero in order to pass test.

^d^ Should not be statistically significant in order to pass test. Cells in bold represents results consistent with predictions of the Balanced Identity model.

#### Supplementary analysis: Comparison with Experiment 1

**Figure 3:**
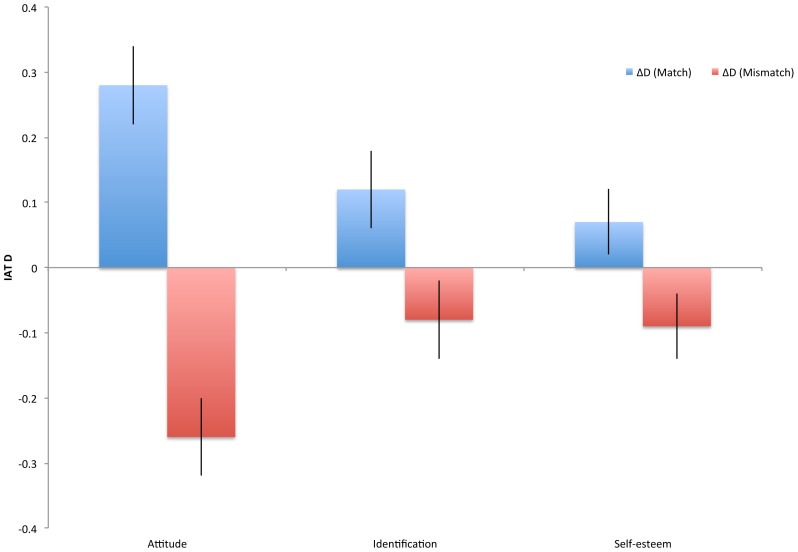
Difference between mean values of Experiment 1 and mean values of the match and mismatch conditions of Experiment 3. Error bars represent standard error of the mean difference; units are the IAT effect size *D.*

As described in Experiment 2, we can estimate the relative degree of change affected by the match versus mismatch condition by comparing to the means from Experiment 1 (no manipulation). As in Experiment 2, the overall means from Experiment 3 (collapsing across conditions) did not differ significantly from those observed in Experiment 1, all *t*<.34, *p*>.74. Results of the comparisons between Experiment 1 means and the two conditions in Experiment 3 are presented in Figure 3. The results again sugest equivalence in the strength of the positive and negative manipulation.

Interestingly, we again observed some cases in which variance appeared to have been reduced by the manipulation, but instead of occurring with respect to the manipulated construct of group attitude, it again appeared only with respect to group identification (in both the match and mismatch conditions). However, as in Experiment 2, the variances between match and mismatch conditions themselves did not differ, suggesting that while manipulation appears to reduce variance, that reduced variance cannot account for the varying degrees of balance observed between match and mismatch conditions.

The results of Experiment 3 lend further support to a causal interpretation of Balanced Identity by showing that intervening on implicit attitudes robustly affected both implicit group identification and self-esteem. Participants whose implicit preference for their ingroup was reduced also implicitly identified with that group less, and even showed less implicit self-related positivity. In addition, and as in Experiment 2, group identity, attitude, and self-esteem remained closely related in the manner predicted by Balanced Identity when group attitude was reinforced, but not when it was disrupted, suggesting that depressing ingroup preference prevents cognitive balance.

## Experiment 4: Manipulating Self-Esteem

A nearly identical procedure to that described in the previous two experiments was employed, except that this time the self-positive association was manipulated. This association differs in one profound respect from the group attitude and group identification associations, in that it is an enduring construct (i.e., implicit self-esteem) that exists before and endures after the experimental paradigm in which participants find themselves. That is, the minimal group paradigm that participants take part in here *produces* group attitude and group identification; it does not *produce* self-esteem in the same way, because the self-valence link is an enduring representation in semantic memory. Does this make it more resistant to the form of manipulation used above? And does it affect the influence it exerts on associated constructs?

### Methods

#### Participants

One hundred sixteen participants were recruited from the same population and following the same procedure described in prior experiments.

#### Procedure

The procedure was closely modeled after that employed in Experiments 2 and 3, except that the manipulation targeted the self-valence association.

#### Manipulation of implicit self-esteem

The same partial-IAT procedure used in prior experiments was used here. That is, as a between-participants factor, participants either repeatedly responded to self and positive words using one key and other and negative words using another key, or repeatedly responded to other and positive words using one key and self and negative words using another key. Thus, the manipulation was designed to either increase or decrease the positive association with the self while affecting the association with other-related concepts in the opposite direction.

#### Measures

The same measures used in Experiment 1 were used here.

### Results and Discussion

#### Descriptive statistics

Standard exclusion criteria for IAT results led to the elimination of data from three participants from the attitude IAT, seven from the self-esteem IAT, and 11 from the identification IAT. There were no effects of ingroup name or task order, and so these factors were dropped from subsequent analysis. Overall results again mirrored those described in the prior experiments, with participants exhibiting robust preference for the ingroup, *D* = .27 (.35), *t*(112)  = 8.27, *p*<.001, *d* = .64, robust identification with the ingroup, *D* = .41 (.36), *t*(104)  = 11.69, *p*<.001, *d* = 1.2, and strong implicit positive self-esteem, *D* = .47 (.35), *t*(109)  = 14.05, *p*<.001, *d* = 1.0. Identification was again stronger than attitude, paired *t*(102)  = 3.05, *p* = .003. Unlike in prior studies, preference for and identification with the ingroup were not correlated, *r*(101)  = .06, *p* = .56, though weak correlations were observed between attitude and self-esteem, *r*(107)  = .20, *p* = .03, and identification and self-esteem, *r*(100)  = .23, *p* = .02.

Participants self-reports also revealed more liking for the ingroup, M = .78 (1.4), *t*(114)  = 6.08, *p*<.001, *d* = .56, as well as greater identification with the ingroup, M  = 2.0 (2.1), *t*(114)  = 10.38, *p*<.001, *d* = .95. Participants also reported somewhat greater liking for the self as compared with others, M = .46 (1.2), *t*(115)  = 4.19, *p*<.001, *d* = .38. Preference and identification were highly correlated, *r*(113)  = .59, *p*<.001, but no other bivariate correlations reached significance, both *r*(113) <.12, *p*>.23. Implicit and explicit identity were moderately correlated, *r*(102)  = .28, *p* = .004, but no other implicit-explicit correlations achieved significance, both |*r*|<.10, *p*>.33.

#### Effect of manipulation

Half of participants completed a manipulation designed to strengthen association between the self and positivity (“match” condition), while the other half completed a manipulation designed to weaken it (“mismatch” condition). This manipulation was effective; participants in the match condition exhibited stronger implicit self-esteem as measured by the IAT, *M_MATCH_* = .56 (.34), *M_MISMATCH_* = .40 (.34), *t*(108)  = 2.53, *p* = .01, *d* = .47, though the size of this effect is considerably smaller than in the previous two experiments, suggesting that self-esteem is indeed more resistant to manipulation in this fashion. The manipulation also affected ingroup preference, *M*_MATCH_ = .36 (.33), *M_MISMATCH_* = .20 (.35), *t*(111)  = 2.39, *p* = .02, *d* = .47, but did not affect ingroup identification, *M*_MATCH_ = .46 (.32), *M_MISMATCH_* = .37 (.39), *t*(103)  = 1.38, *p* = .17, *d* = .25. Positive self-esteem, ingroup identification, and ingroup preference remained statistically significant in both match and mismatch participants, all *t*s>4.5, *p*s<.001. The manipulation had no effect on explicit measures, all *t*<.43, *p*>.67.

#### Balanced Identity Analyses

Table 1 provides a summary of results of the Balanced Identity analyses for implicit and explicit data, collapsing across condition; overall, evidence for balance was strong at the implicit level but weak at the explicit level, with 12 and 6 tests passed, respectively. Table 4 provides these analyses sub-divided by condition (match or mismatch). When broken down by condition the results of this study were somewhat less definitive than in the prior two investigations. For participants in the match condition, implicit data led to 9 of 12 tests being passed, but two of the failures were at Step 1, where for models with identification and self-esteem as criterion, the interaction was directionally consistent but did not reach significance. For explicit data, only 3 of 12 tests were passed, suggesting no balance at this level. In the mismatch condition, there was for the first time some evidence of balance with 10 of 12 tests passed, though again the two failures were at Step 1. For explicit data in the mismatch condition, 6 of 12 tests were passed. In one sense, these data are broadly consistent with prior experiments, in that there was better evidence of balance at the implicit than explicit level. However, evidence of balance was this time as good in the mismatch condition as in the match condition. Additionally, both match and mismatch analyses revealed failures at Step 1, which represent particularly dramatic divergences from predictions.

**Table 4:** Summary of Balanced Identity Analyses for Experiment 4, sub-divided by condition.

			Regression Step							Total tests passed
			Step1			Step 2				
		Criterion (*Y*)	Interaction^a^	*R^2^*	Interaction^b^		Main effect 1^c^	Main effect 2^c^	Δ *R^2^* d	
Experiment 4 (**Match Condition**)	**Implicit**	Attitude	.39*	.09	**.13**		**.15**	**.15**	**.01**	9/12
		Identification	.29	.05	**.12**		**.04**	**.02**	**.01**	
		Self-esteem	.26	.03	**−**.29		**.19**	**.38**	**.04**	
	**Explicit**	Attitude	.11**	.07	**−**.05		**.07**	.54***	.31***	3/12
		Identification	.20***	.09	**−**.12		.81***	.56***	.34***	
		Self-esteem	.02*	.03	**−**.004		**−.003**	.16**	.06*	
Experiment 4 (**Mismatch Condition**)	**Implicit**	Attitude	.19	.02	**.55**		**−.10**	**−.28**	**.03**	10/12
		Identification	.37	.04	**.38**		**−.16**	**.38**	**.06**	
		Self-esteem	.46*	.08	**.36**		**.17**	**−.04**	**.03**	
	**Explicit**	Attitude	.13***	.09	**.05**		**.05**	.37***	.28***	6/12
		Identification	.20*	.05	**.09**		.87***	**−.20**	.31***	
		Self-esteem	.01	.01	**−**.01		**.19**	**−.05**	**.01**	

Notes: Alpha levels are * *p*<.05, ** *p*<.01, *** *p*<.001.

^a^ Should be statistically significant and positive in order to past test.

^b^ Should remain numerically positive in order to pass test.

^c^ Should both not differ statistically from zero in order to pass test.

^d^ Should not be statistically significant in order to pass test. Cells in bold represents results consistent with predictions of the Balanced Identity model.

**Figure 4:**
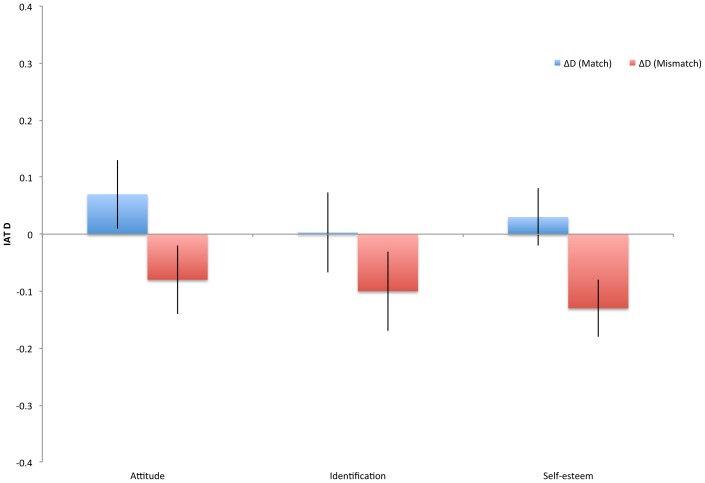
Difference between mean values of Experiment 1 and mean values of the match and mismatch conditions of Experiment 4. Error bars represent standard error of the mean difference; units are the IAT effect size *D.*

One plausible interpretation of the maintenance of balance in the mismatch condition concerns the strength of our manipulation. The manipulation of self-esteem, while statistically significant, was considerably weaker than the manipulation of the other constructs in the previous studies (*d_self-esteem_* = .44, *d_attitude_* = 1.77, *d_identification_* = 1.40; see also Figure 4, introduced below). Thus, the manipulation might not have created a powerful enough perturbation to effect balance as dramatically, perhaps because self-esteem, an enduring and central aspect of personhood, is particularly resistant to negative perturbations.

#### Supplementary analysis: Comparison with Experiment 1

As described in Experiment 2 and 3, above, we again compared results to the means from Experiment 1, which can serve as a no-manipulation baseline. As in the prior two experiments, collapsing cross condition in Experiment 4 reveals means that do not differ significantly from those in Experiment 1, all *t*<1.3, *p*>.20. Results for the comparisons between Experiment 1 and the match and mismatch conditions of Experiment 4 are presented in Figure 4, showing the average change and associated standard errors. Unlike in the previous two experiments, visual inspection of the figure suggests that the manipulation of self-esteem was not particularly effective in the match condition (i.e., the mismatch bars are the only ones that are reliably displaced from 0). Thus, it appears that the manipulation was not powerful enough to inflate already robust implicit self-esteem but was powerful enough to (presumably temporarily) decrease it. As in Experiment 2, results again suggested a (in this case marginal) reduction of variance for the intervened upon construct (self-esteem). But as in the prior experiments, the variances between match and mismatch conditions in Experiment 4 did not differ.

## General Discussion

The current study investigated whether consistency between group attitude, group identification, and self-esteem—which has been widely demonstrated with respect to familiar and salient real-world groups—would emerge immediately following random assignment to minimal social groups. Four experiments provided a strong affirmative answer: cognitive balance was robustly present in every case, but (as has been noted with real-world groups) only at the implicit level. These findings strongly suggest that cognitive balance need not stem from a history of gradual revision or iterative dissonance reduction. Rather, the initial form that intergroup cognitions take is well described by the predictions of Balanced Identity, suggesting that new cognitions are constrained by existing ones as described by the Balance model.

Relatedly, these studies provide direct evidence for a causal (as opposed to merely descriptive) reading of Balanced Identity by showing that manipulating any one leg of the balance triad (e.g., group attitude, group identification, self-esteem) affected related constructs while preserving the broader pattern of cognitive balance. Establishing these causal connections within the framework of the minimal group paradigm supports a reading of Balanced Identity as a generalized causal account of intergroup bias; intergroup bias can be conceptualized as the interactive effects of self-esteem and group identification. What's more, it suggests new pathways to intervention. Intergroup bias can be intervened with not only directly but also by manipulating the two related constructs of group identification and self-esteem.

Finally, the present results bolster the prior observation that belonging to a group that is not positively evaluated inhibits balance for members of that group. When we intervened on group attitude or identification, participants who experienced the “negative” intervention that decreased the strength of that construct did not show cognitive balance. This was not merely the result of any artificial intervention, as participants who experience the “positive” intervention increasing the strength of the same construct did show cognitive balance. Nor was it the result of a reduction in variance in the manipulated construct; while manipulation did appear to reduce variance (as compared to Experiment 1, in which there was no manipulation), it reduced variance equivalently across the match and mismatch conditions and so cannot uniquely explain the different degrees of balance that were then observed. Results of a final experiment manipulating self-esteem were somewhat more equivocal, however, perhaps because the effort to change implicit self-esteem was less successful than the other two manipulations, and the effects it did have may have been asymmetrical, predominately deflecting implicit self-esteem in the negative direction (Figure 4). In any case, one suggestive interpretation of this broader pattern is that, when belonging to a group that is negatively evaluated, balance is disrupted so as to prevent that negativity from bleeding over into self-esteem. While broadly consistent with some motivational accounts of self-group relationships such as Social Identity Theory [24], it is striking that this phenomenon is seen most clearly at the implicit level, where we would expect that motivational concerns would be at least somewhat attenuated. Indeed, a question raised by these findings is how the implicit system “knows” to disrupt balance in the face of a negative perturbation while retaining it in the face of a positive perturbation. The microdynamics of these changes could be a fruitful topic for future study.

Stepping back somewhat, some past research has postulated that intergroup bias is the result of self-related positivity that associatively spreads from the self to ingroups [25]. However, support for this claim has been mixed [26], [27]. The present research suggests that the reason for this mixed support may be that, in trying to associate self-esteem with group bias, one crucial construct, group identification, has been left out. In a sense, it is attempting to impose a bivariate model (i.e., linkages between self-esteem and group attitude) on a multivariate and interactive phenomenon. Looking solely at the link between group attitude and self-esteem in the present studies, the correlations were a modest *r* = .29, and the relationship was significant in some but not other studies. In other words, the same mixed support found in prior investigations appeared in these data. By contrast, when predicting each construct from the product of the other two, implicit data yielded a statistically significant and positive relationship in the great majority of tests across these four studies (with the exception of the cases in which the constructs were artificially reduced through intervention). As with prior work employing the Balanced Identity framework, however, the predicted pattern appears to characterize *implicit* constructs much better than *explicit* constructs. Indeed, as compared with the 47/48 successful statistical tests for implicit data (collapsing for the moment across condition), only 24/48 tests were passed for self-report data. Indeed, it is possible that the relationships between self-report constructs may be better captured by bivariate relationships, which were generally sizable, as compared with the implicit data, which are better-described by the multiplicative relationships specified by Balanced Identity.

One general pattern emerging from my manipulation of individual components of the balance triad was that, while each construct is easily manipulated, the influence of that manipulation on the more “downstream” constructs was not perfectly predictable. That is, it did not always affect *both* theoretically related constructs. Suggestively, the pattern was at least partially structured. Specifically, group attitude and group identification mutually affected one another, and group attitude and self-esteem mutually affected one another. However, self-esteem and group identification did not affect one another in either direction. Future work will be necessary to understand whether this pattern is replicable and, if so, why it occurs.

One potential weakness of the present design is that the method used to manipulate the constructs in question was closely related to the method used to measure them following their manipulation. That is, a partial-IAT procedure was used to manipulate the constructs, which were then measured with a full IAT. While the left/right side pairing was balanced at the participant level such that a simple side-bias is unlikely, it is nonetheless possible that the partial-IAT procedure affected subsequent IATs to some extent. Future work could make the method of manipulation more distinct, for example through a conditioning paradigm, or could alter the method of construct measurement by using a different implicit measure.

In closing, this study highlights the value of the minimal group paradigm as a methodological tool in intergroup social cognition research. By abstracting away from the variable knowledge and informational complexity that characterizes real-world groups, we can gain a clear perspective on how group affiliations emerge and change across contexts and can observe the emergence of such cognitions from their earliest moments. Of course, it is always important to verify that findings with minimal groups generalize to real groups. In the present context, this has already been accomplished, as cognitive balance has been widely observed with respect to real-world groups. But that observation left open several questions regarding the causal status and informational time course of such consistency. The minimal group paradigm became a powerful tool for addressing these issues in a controlled manner. At a higher level, by demonstrating that a phenomenon observed with respect to real-world groups also occurs with respect to novel social groups, we are able to reduce a set of specific observations about specific groups to a general principle governing intergroup cognition more broadly [14]. This opens the door to interpreting Balanced Identity as a general account of intergroup attitudes (as well as, of course, intergroup identifications and self-esteem), offering a model for both prediction and intervention. Future work focusing on linkages between self-esteem and attitudes will benefit by incorporating these insights and ensuring attention is also paid to the closely related construct of group identification.
